# Combined Repeated-Dose Toxicity Study with the Reproduction/Developmental Toxicity Screening Test of Calcium Nitrate Tetrahydrate in Sprague Dawley Rats

**DOI:** 10.3390/toxics13100835

**Published:** 2025-09-30

**Authors:** Ji-Woo Eom, Han-il Kang, Jae-Hyun Lee, Si-Hwan Song, Jeong-hyun Hong, Seungjin Bae, Chun-Ja Nam, Kyung-Min Lim

**Affiliations:** 1 Nonclinical Research Institute, Corestemchemon Inc., 240, Nampyeongro, Yangji-myeon, Cheoin-gu, Yongin-si 17162, Republic of Korea; jweom@csco.co.kr (J.-W.E.); hikang@csco.co.kr (H.-i.K.); jaehlee@csco.co.kr (J.-H.L.); swansong@csco.co.kr (S.-H.S.); 2 Graduate School of Pharmaceutical Sciences, College of Pharmacy, Ewha Womans University, Seoul 03760, Republic of Korea; hongjh126@gmail.com (J.-h.H.); sjbae@ewha.ac.kr (S.B.); 3 Graduate Program in Innovative Biomaterials Convergence, Ewha Womans University, Seoul 03760, Republic of Korea

**Keywords:** repeated-dose toxicity study, reproduction/developmental toxicity screening test, OECD TG 422, calcium nitrate tetrahydrate

## Abstract

Calcium nitrate tetrahydrate, used in fertilizers, wastewater treatment, and concrete admixtures, has limited toxicity data despite extensive industrial use. This study evaluated its repeated-dose and reproductive/developmental toxicity in Sprague Dawley rats following OECD TG 422, which combines TG 407 and 421 to extend dosing than TG 407 and reduce animal use compared with separate studies. Rats were administered 0, 100, 300, or 1000 mg/kg/day. Males were treated for 49 days and females from 2 weeks pre-mating to postpartum day 13; the recovery group was observed for an additional 2 weeks. Endpoints included clinical signs, body weight, food consumption, hematology, serum biochemistry, organ weights, histopathology, reproductive performance, and F1 development. No systemic toxicity was observed in F0 males. Minimal prostate atrophy occurred in high-dose males but was considered non-adverse due to limited severity. One high-dose female died on PPD 1, and high-dose F1 litters showed decreased litter size, increased post-implantation loss, and a reduced live-born index. Based on these results, NOAELs were cautiously assigned 1000 mg/kg/day for repeated-dose and male reproductive toxicity and 300 mg/kg/day for female reproductive and developmental toxicity. TG 422 efficiently characterized hazards while reducing animal use, though its limited duration and scope indicate the need for complementary studies.

## 1. Introduction

Calcium nitrate is widely used in agriculture, construction, and wastewater treatment [1,2], and has applications in cementitious compositions [3,4], advanced concrete technologies such as 3D printing [5,6], and as a coagulant in latex films [7]. Major producers and importers include China, the EU, and the United States [8,9]. In Northern Europe, the reported use of the tetrahydrate form reached up to 4774 tons in Norway in 2021 [10]. Regulatory frameworks, such as EU and Korean REACH, require 28-day repeated-dose and reproduction/developmental screening data for substances produced or imported at volumes ≥ 10 tons/year, with more extensive testing at ≥1000 tons/year. Despite its high production and global trade, no OECD guideline-compliant repeated-dose or reproductive toxicity data for calcium nitrate tetrahydrate have been reported.

Calcium nitrate exists as a tetrahydrate at room temperature and is stable at ranges between 0–30 °C and 30–100% relative humidity [11]. When it dissolves in water, it dissociates into nitrate and calcium ions [12]. Excessive use of calcium nitrate can be harmful to humans and other mammals [13]. Epidemiological studies have reported associations between nitrate exposure in drinking water and adverse reproductive outcomes, including fetal loss, preterm birth, neonatal mortality, and congenital malformations [14,15,16,17]. For example, a nationwide Danish cohort of over one million births showed an elevated risk of preterm birth linked to rising nitrate levels in tap water, even below current regulatory standards [15]. Similarly, U.S. data linking public water nitrate concentrations to birth certificates revealed consistent associations with premature births [16]. A 2022 systematic review further connected nitrate exposure to congenital malformations and preterm delivery [17].

Mechanistic evidence reinforces these findings. Ingested nitrate (NO_3_^−^) undergoes reduction to nitrite (NO_2_^−^), which oxidizes hemoglobin iron (Fe^2+^) to methemoglobin (Fe^3+^), impairing oxygen transport and causing tissue hypoxia [18,19]. This process is particularly critical in infants, as fetal hemoglobin is more readily oxidized and their NADH-dependent reductase systems are immature [18,20]. Genetic vulnerabilities, including G6PD deficiency, further increase susceptibility [20]. Beyond acute hypoxia, chronic nitrate and nitrite exposure have been associated with oxidative stress, red blood cell damage, Heinz body formation, and the endogenous generation of N-nitroso compounds, many of which are known carcinogens [19,20]. Nitrate exposure has also been linked to thyroid hormone imbalance and possible endocrine disruption, though causal relationships remain under investigation [19,20].

Clinical case reports and toxicological studies highlight the risks of excessive nitrate and nitrite exposure. Nitrate and nitrite exposure can also cause smooth muscle relaxation and blood vessel dilation [21], leading to blood pooling in the veins and preventing the heart from filling completely. Methemoglobinemia in infants, often linked to contaminated vegetables or drinking water, remains a classic health concern [22,23]. Severe poisoning can occur at oral nitrate doses >2 g, and the reported lethal dose of nitrite in humans is approximately 4 g [24,25]. These data underscore the importance of evaluating nitrate-containing salts such as calcium nitrate tetrahydrate for potential reproductive, developmental, and systemic effects.

OECD Test Guideline 422 (Combined Repeated-Dose Toxicity Study with the Reproduction/Developmental Toxicity Screening Test) provides a practical framework to address this gap. TG 422 integrates systemic and reproductive/developmental endpoints within a single study, thereby reducing animal use in accordance with the 3Rs principle [26]. It builds upon TG 407 (28-day repeated oral toxicity study) by adding reproductive and developmental assessments and incorporates endpoints adapted from TG 443 (extended one-generation reproductive toxicity study) to detect potential endocrine-disrupting effects (Table S1). While TG 422 is less comprehensive than long-term or multigenerational designs, it provides an efficient screening-level evaluation of hazard potential.

Based on the dissociation of calcium nitrate tetrahydrate into calcium and nitrate ions under physiological conditions, we hypothesized that its toxicological profile would largely reflect nitrate-related mechanisms and that adverse systemic or reproductive/developmental effects would be limited within the applied dosing range. To test this hypothesis, the present study was conducted on Sprague Dawley rats in accordance with OECD Test Guideline 422. The aims of this study were to characterize systemic toxicity and potential target organs after 49 days of repeated oral administration, to assess reproductive performance and early developmental outcomes, and to determine no-observed-adverse-effect levels (NOAELs) for both systemic and reproductive/developmental endpoints. Furthermore, this study sought to demonstrate the practical utility of TG 422 as an efficient hazard screening tool.

## 2. Materials and Methods

### 2.1. Test Substance and Vehicle

Calcium nitrate tetrahydrate (99.6%, CAS No. 13477-34-4) was obtained from Sigma-Aldrich (San Diego, CA, USA). Sterile water for injection, supplied by Dai Han Pharm Co., Ltd. (Seoul, Republic of Korea) was used as the vehicle.

### 2.2. Animals and Husbandry

This study was performed at the Non-clinical Research Institute of Corestemchemon Inc. (Yongin-si, Republic of Korea) in compliance with the Standards and Regulations for Chemical Testing Laboratories (Ministry of Environment, Republic of Korea, Notification No. 2022–9, 11 January 2022) and the OECD Principles of Good Laboratory Practice (1997) ENV/MC/CHEM (98)17. A total of 64 male and 64 female Specific Pathogen-Free (SPF) Sprague Dawley rats (NTacSam: SD) [27,28] were acquired from Sam Tako Bio Korea Co., Ltd. (Osan, Republic of Korea). SD rats were selected due to their common use in general toxicity studies and the availability of substantial reference data facilitating the interpretation and evaluation of the findings. The females were 8 weeks old, and the males were 10 weeks old upon arrival. Animals were examined for physical appearance and weighed upon arrival. Females were acclimatized for 15 days, and males for 8 days. During this period, female estrous cycles were monitored for 14 days to select those with regular cycles. At the start of dosing, the weight range for males was 353.53–411.25 g, and for females it was 201.17–262.95 g. Animals were provided ad libitum access to Teklad certified irradiated global 18% protein rodent diet (Envigo, Indianapolis, IN, USA and Inotiv, West Lafayette, IN, USA), which was supplied by Coretech Co., Ltd. (Pyeongtaek, Republic of Korea). Drinking water was underground water sterilized by ultraviolet irradiation and microfiltration, supplied in polycarbonate bottles, and made available ad libitum. Water quality was tested by the Gyeonggi-do Institute of Health and Environment (Suwon, Republic of Korea) and was compliant with the Korean Drinking Water Standards. Wood bedding was obtained from Central Lab Animal, Inc. (Seoul, Republic of Korea), sterilized by autoclaving, and dried before use. Contaminant analyses were conducted according to the SOP of Corestemchemon Inc., and no factors were identified that could impact the test results. Water was checked daily, and polycarbonate cages, bedding, and water bottles were changed at least once weekly, while stainless-steel cages were changed every two weeks. Cage racks were rotated monthly in a clockwise fashion. Overall, the feed, housing, and environmental conditions were confirmed not to influence the study results.

### 2.3. Study Design

This study was conducted in accordance with the guidelines of OECD TG 422: Combined Repeated-Dose Toxicity Study with the Reproduction/Developmental Toxicity Screening Test (29 July 2016). In compliance with OECD TG 422, the group sizes were selected to perform a reliable evaluation of reproductive and developmental endpoints while limiting the number of animals employed. This study included a vehicle control group and three dosing groups administered 100, 300, and 1000 mg/kg/day of calcium nitrate tetrahydrate. The control and high-dose groups each included 5 male and 5 female recovery animals, for a total of 17 animals. The low-dose and mid-dose groups each comprised 12 male and 12 female animals. Recovery animals remained unmated and underwent a two-week recovery period following the final dose (Table 1). Mating was performed at a 1:1 ratio within groups, confirmed by the detection of a vaginal plug or sperm. For F0 confirmed mated females, delivery was monitored starting on GD 21. The date of delivery was recorded as postpartum day 0 (PPD 0) for the dams and postnatal day 0 (PND 0) for the newborns. All pregnant animals were allowed to deliver naturally. On PND 4, culling was performed to standardize the litter size to 8 pups (4 males and 4 females if possible). The remaining pups were then blood-sampled for T4 measurement. If a litter contained fewer than 8 pups, no culling was performed.

### 2.4. Dose Level Selection

**Figure 1:**
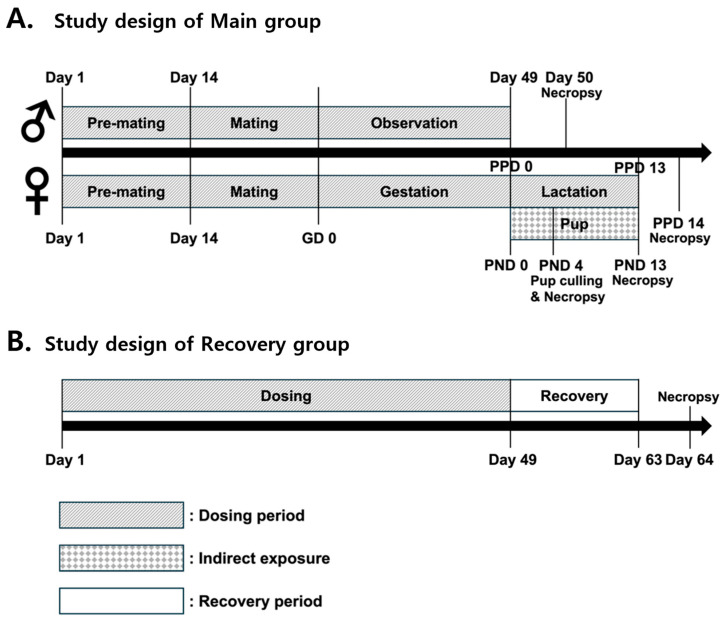
Study design for the combined repeated-dose toxicity study with the reproduction/developmental toxicity screening test of calcium nitrate tetrahydrate in Sprague Dawley rats.

Males in the main group and females in the recovery group were administered the test substance once daily for 49 days starting 2 weeks before cohabitation and continuing until the day before necropsy. Females in the main group were administered the test substance once daily from 2 weeks before cohabitation until post-partum day 13 (PPD 13). Unmated females were administered the test substance daily for 26 days after the final day of cohabitation until the day before necropsy (Figure 1).

### 2.5. Observations and Measurements

- Observations: During the administration and observation periods, animal mortality was monitored, and observations were recorded twice daily. Clinical signs were observed at least once daily. Detailed observations were conducted before the start of dosing and then weekly. Functional observation tests were conducted on five males and five females per group selected from the main group, and on all animals in the recovery group before necropsy. The functional observational battery comprised evaluations of stimulus reactivity (auditory response, righting reflex, nociceptive response, pinna reflex, and threat reflex), neuromuscular performance (grip strength), and locomotor activity outside the home cage, including ambulatory distance, ambulatory count, and vertical count.
- Body Weight: Body weight for all F0 males was measured on day 1, then weekly, and on the day of necropsy. For F0 females, body weight was measured on day 1 and weekly before mating. For confirmed mated females, measurements were conducted on GD 0, 7, 14, and 20; PPD 0, 4, 8, and 13; and on the day of necropsy.
- Food Consumption: Food consumption for main group F0 males and recovery group males and females was recorded on day 1 and weekly thereafter, excluding the cohabitation period. For F0 females, food consumption was measured on day 1 and weekly until the day of cohabitation, and for confirmed mated females on GD 0, 7, 14, and 20 and PPD 0, 4, 8, and 12.
- Reproductive Parameters: To identify the estrous cycle, vaginal smears were performed daily on the main group females from day 1 until mating confirmation. The regularity and duration of the estrous cycle were calculated up to two weeks before the start of cohabitation. Additionally, vaginal smears were collected on the morning of necropsy to determine the stage of the estrous cycle.
- F1 Observation: The number of live and dead newborns was recorded within 24 h of delivery. The sex of live F1 animals was determined to calculate the sex ratio, weights were recorded, and any physical abnormalities were observed. On PND 4, the anogenital distance (AGD) was measured using a caliper, and the anogenital index (AGI) was calculated. On PND 12, the number of nipples/areolas was checked in male offspring. Body weight was measured on PND 0, 4, 8, and 13.
- Clinical Pathology: Hematological and blood biochemical tests were conducted on five males and five females per group from the main group and on all animals in the recovery group. Approximately 1 mL of blood was collected and analyzed by an automated hematology analyzer (ADVIA 2120i, SIEMENS, Munich, Germany) using K2EDTA as an anticoagulant. For coagulation time, plasma was obtained by centrifuging 1.8 mL of blood mixed with 3.2% sodium citrate at a 1:9 ratio (3000 rpm, 800 RCF, Microcentrifuges 5415R, Hamburg, Germany) for 10 min, and the coagulation time was measured using a blood coagulation analyzer (ACL 7000, Instrumentation Laboratory, Bedford, MA, USA). Approximately 2 mL of blood was collected in tubes with a clot activator and left to clot for 15–20 min at room temperature. The samples were then centrifuged for 10 min (3000 rpm, 1902 RCF, Combi-514R, Hanil, Republic of Korea) to obtain serum, which was analyzed using a blood biochemistry analyzer (DxC 700 AU, BECKMAN COULTER, Brea, CA, USA).
- For F1 pups, blood was collected and pooled by litter for PND 4 pups, and by sex within the litter for PND 13 pups. Blood samples were taken from the heart for PND 4 and from the inferior vena cava for PND 13. Approximately 1.2 mL of blood was taken from all F0 animals, and approximately 1 mL from the PND 4 and PND 13 pups. Blood samples were placed in 5 mL Vacutainer tubes with a clot activator, allowed to clot for approximately 30 min at room temperature, and then centrifuged at 3000× *g* (4 °C) for 10 min to obtain serum. Serum samples were stored in a deep freezer (≤−50 °C) to avoid repeated freezing and thawing. Serum analysis, including thyroxine (T4) concentration, was conducted within eight weeks of storage using an ELISA kit (Catalog No. CSB-E05082r, Cusabio, Houston, TX, USA). T4 analysis was performed on the PND 13 F1 generation and all males and females. Since no effects were observed in the PND 13 F1 generation, the test was not performed on PND 4 newborns.
- Necropsy: At necropsy, the weights of the testis, epididymides, prostate gland, and seminal vesicle with coagulating gland of all male animals were measured using an electronic balance (Secura224-1S, Sartorius AG, Göttingen, Germany). The relative weight of each organ to the fasted body weight at necropsy was then calculated. Histopathological examination was performed on tissue slides prepared from fixed organs of five males and five females selected from the vehicle control and high-dose groups, deceased animals, and organs showing visible abnormalities. The examination focused specifically on spermatogenesis and the structure of the testicular interstitial cells. For the histopathological examination, a single transverse section was prepared from the central portion of the ventral lobe of the prostate gland. Lesions were graded using a five-tier severity scale: minimal (the least detectable lesion or very slight change), slight (mild change), moderate (intermediate change), severe (marked change), and massive (very marked change). Histopathological data were processed using the Provantis^®^ program, and diagnostic terminology was based on the Provantis Glossary of INSTEM and INHAND [29,30].
- Data collection and evaluations were performed in a blinded manner to prevent observer bias.

### 2.6. Statistical Analysis

Statistical analysis was performed using the Provantis^®^ system’s statistical software (SAS 9.4). Intergroup comparisons were made using parametric or non-parametric methods, with *p* < 0.05 considered statistically significant. Normality of data for various parameters was analyzed using the Shapiro–Wilks test, and homogeneity of variance was tested using Levene’s test. Intergroup comparisons among the main study groups (G1 vs. G2, G3, and G4) were evaluated using ANOVA and Dunnett’s tests, and recovery group data (G1 vs. G4) were analyzed with Student’s *t*-test. Mating index, fertility index, fecundity index, pregnancy index, gestation index, and observations of newborn appearance were analyzed using Fisher’s exact test. The results were calculated as mean and standard deviation. Histopathological data were analyzed for significance using the chi-squared test, with intergroup differences examined using Fisher’s exact test, which are non-parametric statistical methods.

## 3. Results

### 3.1. Effects on the F0 Main Group and Recovery Group

Dose levels of 100, 300, and 1000 mg/kg/day of calcium nitrate tetrahydrate were selected with reference to the toxicological data obtained from an analog compound: calcium nitrate. In a 30-day repeated oral toxicity study on Wistar rats, calcium nitrate was administered at 200, 400, and 800 mg/kg/day [31]. At 800 mg/kg/day, treatment-related findings included increased relative kidney and liver weights, slight increases in serum ALP and ALT, and histopathological changes, such as hepatocellular degeneration, fatty change, and inflammatory responses. Because calcium nitrate and its tetrahydrate dissociate into the same ions (Ca^2+^ and NO_3_^−^) under physiological conditions, the toxicological profile of the anhydrous form was considered appropriate for informing the dose selection of the tetrahydrate [32,33]. Potential differences related to the hydration state, such as molecular weight adjustment, solubility, and formulation characteristics (e.g., pH or osmolality), were reviewed and controlled to minimize any impact on interpretations. On this basis, 1000 mg/kg/day was established as the high dose for the present study, with 300 and 100 mg/kg/day set as intermediate and low doses at approximately three-fold intervals.

In the 1000 mg/kg/day main group, one female died on PPD 1. Necropsy revealed enlarged adrenal glands, a small thymus, and jejunal autolysis. Histopathological examination revealed uterine hemorrhage in this animal. One stillbirth was observed in the 1000 mg/kg/day female group on PPD 0.

In both the main and control groups, sporadic findings, like fur loss, skin discoloration, and mammary gland induration, were observed, which were considered incidental and unrelated to the test substance due to their infrequent occurrence and lack of a dose–response relationship. No abnormalities related to the test substance were noted in the recovery group. Detailed clinical signs and functional observations showed no effects.

### 3.2. Body Weight and Food Consumption

**Figure 2:**
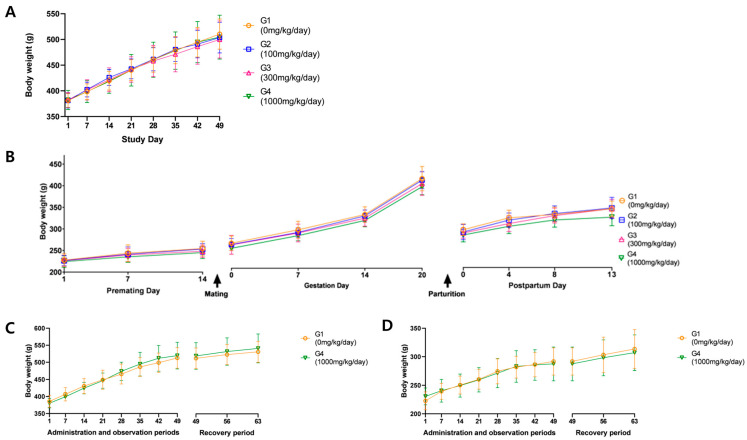
Body weight measurements of (**A**) males in the main group, (**B**) females in the main group, (**C**) males in the recovery group, and (**D**) females in the recovery group. ANOVA and Dunnett tests for (**A**,**B**). *t*-test for (**C**,**D**).

**Figure 3:**
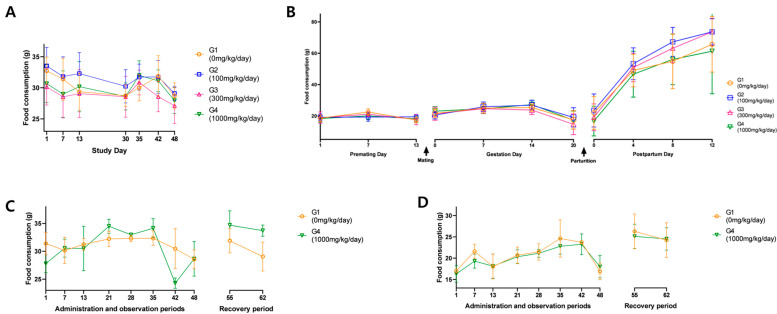
Food consumption of (**A**) males in the main group, (**B**) females in the main group, (**C**) males in the recovery group, and (**D**) females in the recovery group. ANOVA and Dunnett tests for (**A**,**B**). *t*-test for (**C**,**D**).

No substance-related effects on body weight were observed in males of the main group or in males and females of the recovery group. Although females in the 1000 mg/kg/day main group showed a tendency for decreased body weight during pre-mating, pregnancy, and post-mating periods, this was attributed to low body weight on day 1 and was within 10% of the control group’s change. The recovery group showed normal weight gain, suggesting the change was not substance-related (Figure 2). Similarly, food consumption showed no test substance-related effects. Temporary reductions in food consumption were noted in some groups, but these were transient, lacked dose–response relationships, and were not accompanied by body weight changes, and thus considered incidental (Figure 3).

### 3.3. Clinical Pathology

Hematological tests showed no substance-related effects. In the recovery group, males in the 1000 mg/kg/day dose group had a significantly higher mean corpuscular volume (MCV) (*p* < 0.01) and significantly lower red cell distribution width (RDW) (*p* < 0.05). However, the changes were minimal and not observed in the main group, so they were considered unrelated to the test substance. Clinical biochemistry tests showed no substance-related effects. Although some parameters showed statistically significant changes, these were minimal, lacked dose–response relationships, were within the historical control data (HCD) of Corestemchemon Inc. [34], and were not accompanied by related organ changes, indicating they were incidental.

### 3.4. Organ Weights and Histopathology

Absolute and relative thymus weights were significantly lower in males in the 300 and 1000 mg/kg/day dose groups (*p* < 0.05). This was considered a substance effect due to the dose–response correlation. However, the values were within the HCD, and no associated histopathology, hematology, or other immune-related changes were observed in the high-dose group, so it was not considered an adverse effect (Table 2 and Table 3). No substance-related necropsy findings were observed in the recovery group. Although males at 1000 mg/kg/day showed significant increases in the absolute and relative weights of the prostate gland, seminal vesicle with coagulating gland, and other organs, these changes were absent in the main group, lacked histopathological correlates, or were within HCD. In the main group, several sporadic findings were observed across dose groups, but were considered incidental due to rarity, lack of dose–response relationships, and presence in control animals.

Histopathological lesions associated with the test substance were observed in males in the 1000 mg/kg/day dose group. Prostate gland atrophy, with a minimal degree, was observed in three out of five males. In the case of atrophy, glands characterized by reduced epithelial thickness, diminished luminal space, and decreased overall size at the gross level were assessed. When these features were not clearly distinguishable under the microscope, the lesion was classified as minimal. This atrophy was not observed in other dose groups. The prostate gland in the 1000 mg/kg/day group showed no other unusual findings related to the test substance and no effects on reproductive performance, and thus the lesions were considered non-adverse within this study’s context, although their potential biological relevance cannot be fully excluded (Table 4).

### 3.5. Endocrine and Reproductive Parameters

No effects were observed in the main or recovery groups on T4 levels. Although T4 was significantly lower in males in the 300 and 1000 mg/kg/day dose groups (*p* < 0.05, *p* < 0.01), no associated histopathological changes were noted in the thyroid gland, and the T4 concentration remained within the normal range [35] and HCD of Corestemchemon Inc. [34].

No effects were observed on the estrous cycle or reproductive performance. The lower male fertility index in the 300 mg/kg/day group was not considered substance-related due to the lack of a dose–response correlation.

### 3.6. Effect on F1 Pups

**Figure 4:**
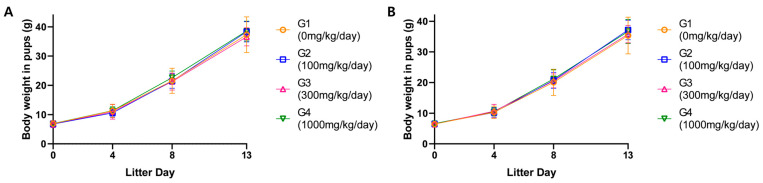
Body weight of (**A**) male pups and (**B**) female pups. ANOVA and Dunnett tests.

No substance-related effects on clinical signs were observed in F1 pups. Cannibalism and deaths were observed in various dose groups, but the lack of a dose–response correlation and presence in the control group suggested these were not caused by the test substance. Hypothermia observed in the 1000 mg/kg/day group occurred in a dam that died the next day and was considered secondary to her deteriorating condition. Body weight of pups showed no substance-related effects (Figure 4).

In the 1000 mg/kg/day dose group, the litter size decreased, post-implantation loss increased, and the live-born index decreased (Table 5).

No effects from the test substance were observed on anogenital distance, number of nipples, or areolas. Necropsy findings in F1 pups were considered incidental. No substance-related effects were observed in T4 levels of the PND 13 pups.

## 4. Discussion

The objective of this test was to determine the reproductive/developmental and 49-day repeated dose toxicity NOAEL of calcium nitrate tetrahydrate by evaluating toxicity, target organs, recovery potential, reproductive/developmental toxicity, and endocrine disruption in Sprague Dawley rats.

One female in the 1000 mg/kg/day dose group was found dead on PPD 1. Necropsy revealed an enlarged adrenal gland, small thymus, and jejunal autolysis, but no clear cause of death was identified. Given the timing of death on the first day after delivery and the observation of uterine hemorrhage, the death was considered to be caused by labor complications and classified as reproductive toxicity. Additionally, a stillbirth was observed in a female from the 1000 mg/kg/day group. This pup mortality, also observed in the high-dose group, was considered to be caused by the test substance and was deemed developmental toxicity. The stillbirth was linked to a low live-born index, as all pups died after birth.

The primary lesion related to the test substance observed in histopathological examination was minimal atrophy of the prostate gland in three out of five males in the 1000 mg/kg/day dose group [36]. Although this was observed in the high-dose group and considered a substance effect, the minimal degree of atrophy [29,37] meant it was not considered an adverse effect. However, such minimal lesions could represent early or subclinical changes, particularly in the context of screening studies, and should therefore be interpreted with caution. Nevertheless, further hormone analyses are needed to clarify the potential involvement of endocrine mechanisms [38].

Regarding litter performance, a decrease in litter size, an increase in post-implantation loss, and a decrease in the live-born index were observed in the high-dose group (1000 mg/kg/day). These changes were considered adverse effects of the test substance and classified as developmental toxicity.

Calcium nitrate exhibits extremely high-water solubility, ensuring nearly complete dissociation into calcium and nitrate ions under physiological conditions [32]. The lethal dose (LD50) was 2000 mg/kg in a single oral toxicity study of polycalcium in Sprague Dawley rats [39], suggesting minimal toxicological effects of calcium salts. This aligns with the findings of studies on other calcium salts. In a 90-day repeated oral toxicity study, nano-calcium carbonate did not induce significant systemic toxicity and showed no differences in serum Ca^2+^ concentrations compared to the vehicle control. Based on these findings, the no-observed-adverse-effect level (NOAEL) of nano-calcium carbonate was determined to be 1000 mg/kg/day in SD rats [40]. These results collectively indicate that calcium salts exert minimal toxicological effects, supporting their continued use with low health risk, which is consistent with the physiological regulation of calcium homeostasis. In mammals, excess calcium is tightly controlled through absorption in the intestine [41], storage and buffering in bone [42], and excretion via renal pathways [43], thereby preventing systemic accumulation and toxicity under normal conditions.

In contrast, nitrate is rapidly absorbed in the upper small intestine and widely distributed, with approximately 60–70% excreted unchanged in urine [44]. A significant fraction enters the entero-salivary circulation, where it is secreted into saliva and reduced to nitrite by oral microbiota [18]. Nitrite can oxidize hemoglobin iron (Fe^2+^) to methemoglobin (Fe^3+^), thereby impairing oxygen delivery and leading to tissue hypoxia, particularly in infants with immature reductase systems. Moreover, nitrite may react with amines to form N-nitroso compounds, many of which are recognized carcinogens [20]. Epidemiological studies have further linked nitrate exposure to reproductive and developmental issues, including fetal loss, neonatal mortality, preterm birth, and congenital malformations [14,15,16,17,20]. Taken together, these mechanistic and toxicokinetic insights strongly support that the adverse reproductive and developmental findings observed with calcium nitrate tetrahydrate in the present study are driven by the nitrate component, rather than by calcium itself.

OECD TG 422 involves administration for two weeks during premating, for at least one day to a maximum of three weeks during mating, and for two weeks after mating. In contrast, OECD TG 407 requires administration for four weeks. Additionally, OECD TG 407 must begin before the animals reach nine weeks of age whereas OECD TG 421 and 422 start after the animals have reached sexual maturity at 10 weeks of age. According to the minimum animal numbers specified in the guidelines, OECD TG 422 requires 108 animals. When OECD TG 407 and 421 are conducted separately, 60 and 88 animals are required, respectively, thereby totaling 148 animals. We demonstrated that TG 422 is an efficient method for hazard characterization, reducing animal use by approximately 27% compared to separate TG 407 and TG 421 studies while providing comprehensive systemic and reproductive toxicity data.

This study was conducted in accordance with OECD Test Guideline 422, which integrates repeated-dose and reproductive/developmental toxicity endpoints to provide screening data while minimizing animal use [45]. Nevertheless, several methodological limitations inherent to this design warrant consideration. First, the relatively short duration of exposure constrains the ability to draw conclusions regarding chronic toxicity or long-term reproductive outcomes. Second, although the number of animals per group aligns with the guideline requirements, the statistical power remains limited for detecting subtle alterations or rare histopathological findings. Feasibility analyses of TG 421/422 endpoints have indicated that while this design is sufficient to capture moderate-to-large effects, minor changes may remain undetected [45]. Third, although a reduction in thymus weight was recorded, functional immune assays such as T-cell-dependent antibody response or NK cell activity were not conducted. Since thymus weight alone is a sensitive but non-specific marker, the absence of complementary functional data limits the ability to conclude on immunotoxic potential [46]. Finally, the evaluation of offspring was limited to early postnatal development, with no data on later-life reproductive capacity, growth, or neurobehavioral outcomes, which are more comprehensively assessed in extended one-generation reproductive toxicity studies (OECD TG 443) [47]. Consequently, subtle or long-term effects such as neurodevelopmental defects [48] cannot be entirely excluded, underscoring the need for additional studies to ensure a comprehensive hazard characterization.

In summary, the reproductive toxicity NOAEL (no observed adverse effect level) of calcium nitrate tetrahydrate is determined to be 1000 mg/kg/day for males and 300 mg/kg/day for females. The developmental toxicity NOAEL is 300 mg/kg/day. NOAEL for repeated-dose toxicity for 49 days is estimated to be 1000 mg/kg/day. These assignments were made with caution, considering the maternal death, reduced litter size, increased post-implantation loss, and decreased live-born index observed at 1000 mg/kg/day. We also demonstrated that OECD TG 422 proved to be efficient, reducing animal use compared to separate TG 407 and 421 studies, and provided robust hazard characterization data.

## Figures and Tables

**Table 1:** Test group composition and administration.

Group	Sex	Number of Animals	Administration Volume (mL/kg/Day)	Dosage of Calcium Nitrate Tetrahydrate (mg/kg/Day)
G1	M/F	17/17	5	0
G2	M/F	12/12	5	100
G3	M/F	12/12	5	300
G4	M/F	17/17	5	1000

**Table 2:** Absolute and relative organ weights of main group. Data are expressed as mean ± SD.

Males in Main Group					Females in Main Group				
Group	G1 0 mg/kg/Day	G2 100 mg/kg/Day	G3 300 mg/kg/Day	G4 1000 mg/kg/Day	Group	G1 0 mg/kg/Day	G2 100 mg/kg/Day	G3 300 mg/kg/Day	G4 1000 mg/kg/Day
Terminal BW (g)	479.50 ± 28.64	472.34 ± 27.12	471.29 ± 34.22	473.48 ± 40.87	Terminal BW (g)	311.31 ± 6.75	306.61 ± 12.89	304.67 ± 14.74	305.68 ± 11.17
Adrenal gland_L wt. (g)	0.0309 ± 0.0031	0.0286 ± 0.0038	0.0265 ± 0.0021	0.0259 ± 0.0034	Adrenal gland_L wt. (g)	0.0408 ± 0.0030	0.0404 ± 0.0083	0.0388 ± 0.0034	0.0418 ± 0.0049
Adrenal gland_L (%)	0.0064 ± 0.0006	0.0060 ± 0.0004	0.0054 ± 0.0005	0.0056 ± 0.0008	Adrenal gland_L (%)	0.0131 ± 0.0007	0.0131 ± 0.0024	0.0128 ± 0.0015	0.0136 ± 0.0013
Adrenal gland_R wt. (g)	0.0291 ± 0.0030	0.0278 ± 0.0043	0.0265 ± 0.0031	0.0252 ± 0.0037	Adrenal gland_R wt. (g)	0.0409 ± 0.0033	0.0382 ± 0.0071	0.0366 ± 0.0049	0.0398 ± 0.0022
Adrenal gland_R (%)	0.0061 ± 0.0006	0.0058 ± 0.0006	0.0054 ± 0.0007	0.0054 ± 0.0008	Adrenal gland_R (%)	0.0131 ± 0.0009	0.0124 ± 0.0021	0.0120 ± 0.0013	0.0130 ± 0.0008
Pituitary gland wt. (g)	0.0128 ± 0.0013	0.0144 ± 0.0015	0.0142 ± 0.0015	0.0144 ± 0.0021	Pituitary gland wt. (g)	0.0144 ± 0.0018	0.0148 ± 0.0019	0.0144 ± 0.0014	0.0153 ± 0.0019
Pituitary gland (%)	0.0027 ± 0.0002	0.0030 ± 0.0004	0.0029 ± 0.0003	0.0031 ± 0.0007	Pituitary gland (%)	0.0046 ± 0.0005	0.0048 ± 0.0006	0.0047 ± 0.0004	0.0050 ± 0.0006
Thymus wt. (g)	0.4438 ± 0.0987	0.3411 ± 0.0492	0.3297 ± 0.0738 *	0.3014 ± 0.0274 *	Thymus wt. (g)	0.2698 ± 0.0443	0.2496 ± 0.0608	0.2487 ± 0.0543	0.2410 ± 0.0226
Thymus (%)	0.0927 ± 0.0218	0.0713 ± 0.0092	0.0671 ± 0.0125 *	0.0651 ± 0.0082 *	Thymus (%)	0.0868 ± 0.0152	0.0812 ± 0.0184	0.0818 ± 0.0187	0.0788 ± 0.0057
PG + SVCG wt. (g)	3.6141 ± 0.5058	3.5827 ± 0.6173	3.1934 ± 0.05024	3.2280 ± 0.4602	Uterus and cervix wt. (g)	0.5025 ± 0.1166	0.4476 ± 0.0651	0.5442 ± 0.1145	0.4844 ± 0.1817
PG + SVCG (%)	0.7549 ± 0.1030	0.7581 ± 0.1199	0.6795 ± 0.1060	0.6873 ± 0.1223	Uterus and cervix (%)	0.1616 ± 0.0385	0.1464 ± 0.0245	0.1797 ± 0.0439	0.1592 ± 0.0624
Testis_L wt. (g)	2.0383 ± 0.1333	2.0922 ± 0.1762	2.0316 ± 0.1960	2.0961 ± 0.1399	Ovary_L wt. (g)	0.0433 ± 0.0081	0.0468 ± 0.0037	0.0439 ± 0.0045	0.0432 ± 0.0076
Testis_L (%)	0.4265 ± 0.0374	0.4440 ± 0.0428	0.4322 ± 0.0413	0.4448 ± 0.0377	Ovary_L (%)	0.0139 ± 0.0024	0.0152 ± 0.0008	0.0145 ± 0.0021	0.0141 ± 0.0023
Testis_R wt. (g)	2.0340 ± 0.1386	1.9737 ± 0.3152	1.9949 ± 0.1807	2.0810 ± 0.1263	Ovary_R wt. (g)	0.0450 ± 0.0074	0.0478 ± 0.0084	0.0502 ± 0.0109	0.0529 ± 0.0072
Testis_R (%)	0.4260 ± 0.0432	0.4182 ± 0.0657	0.4245 ± 0.0391	0.4416 ± 0.0358	Ovary_R (%)	0.0144 ± 0.0021	0.0157 ± 0.0031	0.0165 ± 0.0036	0.0173 ± 0.0025
Epididymis_L wt. (g)	0.7432 ± 0.0516	0.7565 ± 0.0721	0.7165 ± 0.0612	0.7195 ± 0.0438	-	-	-	-	-
Epididymis_L (%)	0.1555 ± 0.0136	0.1604 ± 0.0155	0.1523 ± 0.0115	0.1529 ± 0.0157	-	-	-	-	-
Epididymis_R wt. (g)	0.7524 ± 0.0415	0.7152 ± 0.1444	0.7112 ± 0.0640	0.7319 ± 0.0391	-	-	-	-	-
Epididymis_R (%)	0.1574 ± 0.0129	0.1514 ± 0.0303	0.1511 ± 0.0113	0.1554 ± 0.0122	-	-	-	-	-
Spleen wt. (g)	0.8820 ± 0.0663	0.8966 ± 0.1007	0.8894 ± 0.1387	0.7461 ± 0.0712	Spleen wt. (g)	0.7496 ± 0.1134	0.6913 ± 0.0455	0.7541 ± 0.0870	0.7110 ± 0.1614
Spleen (%)	0.1838 ± 0.0142	0.1874 ± 0.0176	0.1810 ± 0.0283	0.1605 ± 0.0109	Spleen (%)	0.2407 ± 0.0353	0.2255 ± 0.0109	0.2478 ± 0.0302	0.2318 ± 0.0460
Kidney_L wt. (g)	1.4103 ± 0.1681	1.4932 ± 0.1427	1.5616 ± 0.1391	1.5227 ± 0.1413	Kidney_L wt. (g)	0.9829 ± 0.0957	0.9819 ± 0.0813	0.9767 ± 0.0660	1.0059 ± 0.0437
Kidney_L (%)	0.2932 ± 0.0277	0.3125 ± 0.0286	0.3179 ± 0.0303	0.3273 ± 0.0188	Kidney_L (%)	0.3159 ± 0.0321	0.3204 ± 0.0265	0.3205 ± 0.0134	0.3290 ± 0.0055
Kidney_R wt. (g)	1.5224 ± 0.1069	1.5561 ± 0.1655	1.5476 ± 0.1395	1.5680 ± 0.1836	Kidney_R wt. (g)	1.0197 ± 0.0899	0.9784 ± 0.0756	0.9968 ± 0.0489	1.0174 ± 0.0494
Kidney_R (%)	0.3172 ± 0.0234	0.3255 ± 0.0309	0.3151 ± 0.0309	0.3365 ± 0.0235	Kidney_R (%)	0.3279 ± 0.0327	0.3192 ± 0.0243	0.3275 ± 0.0175	0.3328 ± 0.0088
Heart wt. (g)	1.5379 ± 0.1105	1.5517 ± 0.0977	1.5254 ± 0.0209	1.5639 ± 0.1571	Heart wt. (g)	1.1606 ± 0.0708	1.1644 ± 0.0602	1.1905 ± 0.0692	1.1210 ± 0.0383
Heart (%)	0.3201 ± 0.0163	0.3250 ± 0.0219	0.3104 ± 0.0050	0.3371 ± 0.0348	Heart (%)	0.3727 ± 0.0192	0.3805 ± 0.0283	0.3911 ± 0.0226	0.3669 ± 0.0104
Lung wt. (g)	1.8082 ± 0.1166	1.7926 ± 0.1733	1.7605 ± 0.0836	1.7693 ± 0.1365	Lung wt. (g)	1.4380 ± 0.1226	1.4259 ± 0.0886	1.4075 ± 0.0765	1.5231 ± 0.0845
Lung (%)	0.3767 ± 0.0221	0.3745 ± 0.0226	0.3583 ± 0.0180	0.3805 ± 0.0157	Lung (%)	0.4616 ± 0.0325	0.4650 ± 0.0185	0.4626 ± 0.0290	0.4988 ± 0.0323
Brain wt. (g)	2.1803 ± 0.0803	2.1608 ± 0.1182	2.1550 ± 0.0947	2.1885 ± 0.0601	Brain wt. (g)	2.0150 ± 0.0787	1.9885 ± 0.0753	1.9805 ± 0.0129	1.9990 ± 0.0399
Brain (%)	0.4549 ± 0.0301	0.4531 ± 0.0366	0.4386 ± 0.0216	0.4725 ± 0.0357	Brain (%)	0.6475 ± 0.0287	0.6494 ± 0.0350	0.6512 ± 0.0308	0.6547 ± 0.0298
Liver wt. (g)	12.9097 ± 1.1258	13.5829 ± 0.8437	14.2595 ± 0.9945	12.1752 ± 1.5089	Liver wt. (g)	11.3336 ± 0.7904	11.3366 ± 1.3191	11.0026 ± 0.9619	11.2929 ± 0.7646
Liver (%)	2.6854 ± 0.1568	2.8427 ± 0.1476	2.9005 ± 0.1783	2.6129 ± 0.1943	Liver (%)	3.6405 ± 0.2398	3.6932 ± 0.3474	3.6066 ± 0.1532	3.6926 ± 0.1706
Thyroid gland + PTG_L (g)	0.0120 ± 0.0016	0.0138 ± 0.0025	0.0132 ± 0.0036	0.0121 ± 0.0020	Thyroid gland + PTG_L (g)	0.0096 ± 0.0019	0.0085 ± 0.0016	0.0089 ± 0.0017	0.0099 ± 0.0011
Thyroid gland + PTG_L (%)	0.0025 ± 0.0004	0.0029 ± 0.0004	0.0027 ± 0.0027	0.0026 ± 0.0004	Thyroid gland + PTG_L (%)	0.0031 ± 0.0005	0.0028 ± 0.0005	0.0029 ± 0.0007	0.0032 ± 0.0004
Thyroid gland + PTG_R (g)	0.0120 ± 0.0019	0.0130 ± 0.0017	0.0134 ± 0.0016	0.0114 ± 0.0017	Thyroid gland + PTG_R (g)	0.0096 ± 0.0022	0.0085 ± 0.0014	0.0088 ± 0.0015	0.0105 ± 0.0011
Thyroid gland + PTG_R (%)	0.0025 ± 0.0004	0.0027 ± 0.0003	0.0027 ± 0.0003	0.0025 ± 0.0005	Thyroid gland + PTG_R (%)	0.0031 ± 0.0006	0.0028 ± 0.0004	0.0029 ± 0.0005	0.0034 ± 0.0004

ANOVA and Dunnett tests: * = *p* < 0.05.

**Table 3:** Absolute and relative organ weights of recovery group. Data are expressed as mean ± SD.

Males in Recovery Group			Females in Recovery Group		
Group	G1 0 mg/kg/Day	G4 1000 mg/kg/Day	Group	G1 0 mg/kg/Day	G4 1000 mg/kg/Day
Terminal BW (g)	498.70 ± 30.89	506.56 ± 37.24	Terminal BW (g)	289.93 ± 32.80	283.09 ± 29.16
Adrenal gland_L wt. (g)	0.0232 ± 0.0013	0.0269 ± 0.0040	Adrenal gland_L wt. (g)	0.0354 ± 0.0028	0.0373 ± 0.0018
Adrenal gland_L (%)	0.0047 ± 0.0003	0.0054 ± 0.0010	Adrenal gland_L (%)	0.0124 ± 0.0021	0.0133 ± 0.0015
Adrenal gland_R wt. (g)	0.0236 ± 0.0020	0.0256 ± 0.0049	Adrenal gland_R wt. (g)	0.0339 ± 0.0043	0.0350 ± 0.0017
Adrenal gland_R (%)	0.0047 ± 0.0003	0.0051 ± 0.0011	Adrenal gland_R (%)	0.0119 ± 0.0024	0.0125 ± 0.0018
Pituitary gland wt. (g)	0.0117 ± 0.0010	0.0133 ± 0.0005 **	Pituitary gland wt. (g)	0.0165 ± 0.0010	0.0159 ± 0.0014
Pituitary gland (%)	0.0023 ± 0.0002	0.0026 ± 0.0002	Pituitary gland (%)	0.0058 ± 0.0008	0.0057 ± 0.0008
Thymus wt. (g)	0.4143 ± 0.1163	0.3252 ± 0.0417	Thymus wt. (g)	0.2308 ± 0.0348	0.3188 ± 0.0384 **
Thymus (%)	0.0827 ± 0.0212	0.0641 ± 0.0057	Thymus (%)	0.0807 ± 0.0157	0.1131 ± 0.0127 **
PG + SVCG wt. (g)	2.3470 ± 0.2951	3.6049 ± 0.3585 ***	Uterus and cervix wt. (g)	0.9778 ± 0.4308	0.7905 ± 0.2977
PG + SVCG (%)	0.4730 ± 0.0711	0.7170 ± 0.1125 **	Uterus and cervix (%)	0.3524 ± 0.1831	0.2832 ± 0.1093
Testis_L wt. (g)	2.0015 ± 0.1004	2.0155 ± 0.0739	Ovary_L wt. (g)	0.0461 ± 0.0058	0.0468 ± 0.0062
Testis_L (%)	0.4019 ± 0.0187	0.3993 ± 0.0279	Ovary_L (%)	0.0162 ± 0.0034	0.0166 ± 0.0025
Testis_R wt. (g)	1.9811 ± 0.1104	2.0479 ± 0.0567	Ovary_R wt. (g)	0.0442 ± 0.0034	0.0473 ± 0.0071
Testis_R (%)	0.3978 ± 0.0218	0.4060 ± 0.0311	Ovary_R (%)	0.0153 ± 0.0009	0.0170 ± 0.0039
Epididymis_L wt. (g)	0.6883 ± 0.0270	0.7806 ± 0.0708 *	-		
Epididymis_L (%)	0.1384 ± 0.0097	0.1548 ± 0.0184	-		
Epididymis_R wt. (g)	0.6942 ± 0.0539	0.7704 ± 0.0589	-		
Epididymis_R (%)	0.1397 ± 0.0146	0.1530 ± 0.0186	-		
Spleen wt. (g)	0.8844 ± 0.0898	0.9037 ± 0.1329	Spleen wt. (g)	0.7271 ± 0.2173	0.6643 ± 0.0444
Spleen (%)	0.1772 ± 0.0111	0.1778 ± 0.0137	Spleen (%)	0.2486 ± 0.0581	0.2360 ± 0.0195
Kidney_L wt. (g)	1.4099 ± 0.0697	1.5381 ± 0.1131	Kidney_L wt. (g)	0.9120 ± 0.0357	0.9410 ± 0.0530 5
Kidney_L (%)	0.2830 ± 0.0096	0.3038 ± 0.0105 *	Kidney_L (%)	0.3167 ± 0.0239	0.3343 ± 0.0269
Kidney_R wt. (g)	1.4710 ± 0.0817	1.5768 ± 0.1810	Kidney_R wt. (g)	0.8997 ± 0.0448	0.9451 ± 0.0401
Kidney_R (%)	0.2954 ± 0.0152	0.3108 ± 0.0180	Kidney_R (%)	0.3126 ± 0.0268	0.3364 ± 0.0332
Heart wt. (g)	1.5544 ± 0.1630	1.5011 ± 0.1254	Heart wt. (g)	1.0002 ± 0.0741	1.0127 ± 0.0580
Heart (%)	0.3115 ± 0.0239	0.2971 ± 0.0267	Heart (%)	0.3466 ± 0.0224	0.3602 ± 0.0346
Lung wt. (g)	1.8120 ± 0.0843	1.7842 ± 0.0962	Lung wt. (g)	1.3826 ± 0.0844	1.3869 ± 0.0920
Lung (%)	0.3642 ± 0.0231	0.3527 ± 0.0113	Lung (%)	0.4811 ± 0.0535	0.4925 ± 0.0380
Brain wt. (g)	2.1296 ± 0.0220	2.1865 ± 0.0851	Brain wt. (g)	1.9620 ± 0.0890	2.0313 ± 0.1085
Brain (%)	0.4283 ± 0.0266	0.4331 ± 0.0297	Brain (%)	0.6815 ± 0.0565	0.7227 ± 0.0698
Liver wt. (g)	13.7798 ± 1.9023	13.5937 ± 2.2010	Liver wt. (g)	7.4464 ± 0.9400	7.0206 ± 0.5055
Liver (%)	2.7544 ± 0.2307	2.6718 ± 0.2377	Liver (%)	2.5678 ± 0.1359	2.4899 ± 0.1569
Thyroid gland + PTG_L (g)	0.0122 ± 0.0018	0.0128 ± 0.0025	Thyroid gland + PTG_L (g)	0.0088 ± 0.0016	0.0095 ± 0.0010
Thyroid gland + PTG_L (%)	0.0025 ± 0.0004	0.0025 ± 0.0005	Thyroid gland + PTG_L (%)	0.0031 ± 0.0007	0.0034 ± 0.0007
Thyroid gland + PTG_R (g)	0.0125 ± 0.0021	0.0131 ± 0.0018	Thyroid gland + PTG_R (g)	0.0090 ± 0.0010	0.0091 ± 0.0009
Thyroid gland + PTG_R (%)	0.0025 ± 0.0005	0.0026 ± 0.0003	Thyroid gland + PTG_R (%)	0.0032 ± 0.0006	0.0033 ± 0.0006

*T*-test: * = *p* < 0.05; ** = *p* < 0.01; *** = *p* < 0.001.

**Table 4:** Histopathological observation of the prostate glands of male SD rats.

Finding	Main (mg/kg/Day)				Recovery (mg/kg/Day)	
	G1 (0)	G2 (100)	G3 (300)	G4 (1000)	G1 (0)	G4 (1000)
Atrophy of prostate gland	0	0	0	3	0	0
Minimal (+1)	0	0	0	3	0	0

Number of animals.

**Table 5:** Litter performance. Data are expressed as mean ± SD.

Group	G1 0 mg/kg/Day	G2 100 mg/kg/Day	G3 300 mg/kg/Day	G4 1000 mg/kg/Day
No. of pregnant dams	10	12	10	10
No. of dead pregnancy	0	0	0	1
No. of stillbirth	0	0	0	1
No. of litters with live-born pups	10	12	10	9
Gestation length (days)	22.60 ± 0.52	22.33 ± 0.49	22.30 ± 0.48	22.50 ± 0.53
No. of corpora lutea	16.60 ± 1.65	17.50 ± 1.45	16.20 ± 3.65	15.30 ± 1.77
No. of implantation sites	16.60 ± 1.65	17.50 ± 1.45	16.20 ± 3.65	15.30 ± 1.77
Pre-implantation loss (%)	0.00 ± 0.00	0.00 ± 0.00	0.00 ± 0.00	0.00 ± 0.00
No. of total pups born	148	197	155	148
No. of live pups (PND 0)	145	195	153	120
Litter size	14.50 ± 3.75	16.25 ± 1.76	15.30 ± 4.19	12.00 ± 5.58
Sex ratio (% males)	52.39 ± 12.45	53.49 ± 15.67	50.67 ± 17.16	53.78 ± 14.81
Post-implantation loss (%)	13.23 ± 19.18	7.12 ± 6.80	6.58 ± 8.79	20.52 ± 35.67
Dead pups (PND 0)	0.30 ± 0.67	0.17 ± 0.39	0.20 ± 0.63	2.80 ± 5.88
Live pups precull (PND 4)	12.70 ± 5.91	16.17 ± 1.70	14.50 ± 3.72	10.56 ± 6.21
Live pups postcull (PND 4)	7.67 ± 1.00	8.00 ± 0.00	7.90 ± 0.32	8.00 ± 0.00
Live pups on day 13	7.67 ± 1.00	8.00 ± 0.00	7.90 ± 0.32	8.00 ± 0.00
Litters with dead pups (%)	20.0	16.7	10.0	30.0
Litters with ex. abnormalities (%)	0.0	0.0	0.0	0.0
Pups with ex. anomalies (%)	0.0	0.0	0.0	0.0
Gestation index (%)	100.0	100.0	100.0	90.0
Live born index (%)	98.23 ± 3.81	98.98 ± 2.39	99.05 ± 3.01	82.14 ± 36.46
Viability index (%)	87.78 ± 31.30	99.54 ± 1.60	95.70 ± 7.92	83.85 ± 34.43

ANOVA and Dunnett tests.

## Data Availability

Data are contained within the article. The HCD data used in this study are internal data owned by Corestemchemon Inc. and are not publicly available due to confidentiality restrictions.

## Supplementary Materials

The following supporting information can be downloaded at: https://www.mdpi.com/article/10.3390/toxics13100835/s1, Table S1. Procedure of Toxicity Studies mentioned in OECD Guideline 422, 421 and 407 [1].
