# Supplementary material for: Combined Repeated-Dose Toxicity Study with the Reproduction/Developmental Toxicity Screening Test of Calcium Nitrate Tetrahydrate in Sprague Dawley Rats
Source: Toxics. 2025 Sep 30;13(10):835. doi: 10.3390/toxics13100835 (PMC12567573; doi:10.3390/toxics13100835)
Supplement: Supplementary file 1 [file toxics-13-00835-s001.zip › toxics-3876522-supplementary.pdf]

Supplementary Table S1. Procedure of Toxicity Studies mentioned in OECD Guideline 422, 421 and 407 [1]

OECD Guideline	TG 422	TG 407	TG 421
Purpose of study	This Guideline is designed to generate limited information concerning the effects of a test chemical on male and female reproductive performance such as gonadal function, mating behavior, conception, development of the conceptus and parturition.	This TG is intended to investigate effects on a very broad variety of potential targets of toxicity. It provides information on the possible health hazards likely to arise from repeated exposure over a relatively limited period of time, including effects on the nervous, immune and endocrine systems.	This Guideline is designed to generate limited information concerning the effects of a test chemical on male and female reproductive performance such as gonadal function, mating behavior, conception, development of the conceptus and parturition.
Information	The basic repeated dose toxicity study that may be used for chemicals on which a 90-day study is not warranted (e.g. when the production volume does not exceed certain limits) or as a preliminary study to a long-term study.	The basic repeated dose toxicity study that may be used for chemicals on which a 90-day study is not warranted (e.g. when the production volume does not exceed certain limits) or as a preliminary study to a long-term study.	This test can be used to provide initial information on possible effects on reproduction and/or development, either at an early stage of assessing the toxicological properties of chemicals, or on chemicals of concern. It can also be used as part of a set of initial screening tests for existing chemicals for which little or no toxicological information is available, as a dose range finding study for more extensive reproduction / developmental studies, or when otherwise considered relevant.
Comparison	The dosing period is longer than in a conventional 28-day repeated dose study. However, it needs fewer animals of each sex per group when compared with the situation where a stand-alone 28-day repeated dose study is conducted in addition to a Reproduction/Developmental Toxicity Screening Test.	-	-
Number and sex of animals	It is recommended that each group be started with at least 10 males and 12-13 females. Females will be evaluated pre-exposure for estrous cyclicity and animals that fail to exhibit typical 4-5 day cycles will not be included in the study; therefore, extra females are recommended in order to yield 10 females per group. Except in the case of marked toxic effects, it is expected that this will provide at least 8 pregnant females per	At least 10 animals (five female and five male) should be used at each dose level. Consideration should be given to an additional satellite group of ten animals (five per sex) in the control and in the top dose group for observation of reversibility, persistence, or delayed	It is recommended that each group be started with at least 10 males and 12-13 females. Females will be evaluated pre-exposure for oestrous cyclicity and animals that fail to exhibit typical 4-5 day cycles will not be included in the study; therefore, extra females are recommended in order to yield 10 females per group. Except in the case of marked toxic effects, it is expected that

	group which normally is the minimum acceptable number of pregnant females per group. Consideration should be given to an additional satellite group of five animals per sex in the control and the top dose group for observation of reversibility, persistence or delayed occurrence of systemic toxic effects, for at least 14 days post treatment.	occurrence of toxic effects, for at least 14 days post treatment.	this will provide at least 8 pregnant females per group which normally is the minimum acceptable number of pregnant females per group.
Age of animals	Dosing of both sexes should begin 2 weeks prior to mating, after they have been acclimatised for at least five days and females have been screened for normal estrous cycles (in a 2 weeks pre-treatment period). The study should be scheduled in such a way that estrous cycle evaluation begins soon after the animals have attained full sexual maturity. This may vary slightly for different strains of rats in different laboratories, e.g. Sprague Dawley rats 10 weeks of age, Wistar rats about 12 weeks of age.	Dosing should begin as soon as feasible after weaning, and, in any case, before the animals are nine weeks old.	Dosing of both sexes should begin 2 weeks prior to mating, after they have been acclimatised for at least five days and females have been screened for normal estrous cycles (in a 2 weeks pre-treatment period). The study should be scheduled in such a way that estrous cycle evaluation begins soon after the animals have attained full sexual maturity. This may vary slightly for different strains of rats in different laboratories, e.g. Sprague Dawley rats 10 weeks of age, Wistar rats about 12 weeks of age.
Hematology[1]	Five males and five females randomly selected from each group.	All animals	-
Clinical biochemistry[2]	Blood samples obtained from the selected five males and five females of each group, at least two pups per litter on day 4 after birth, if the number of pups allows, and all dams and at least two pups per litter at termination on day 13, and all adult males, at termination.	Blood samples obtained from the selected.	-
T4	Blood samples from the day 13 pups and the adult males are assessed for serum levels for thyroid hormones (T4). Further assessment of T4 in blood samples from the dams and day 4 pups is done if relevant. As an option, other hormones may be measured if relevant. Pup blood can be pooled by litter for thyroid hormone analyses. Thyroid hormones (T4 and TSH) should preferably be measured as 'total'.	Although in the international evaluation of the endocrine related endpoints a clear advantage for the determination of thyroid hormones (T3, T4) and TSH could not be demonstrated, it may be helpful to retain plasma or serum samples to measure T3, T4 and TSH (optional) if there is an indication for an effect on the pituitary-thyroid axis	Blood samples from the day 13 pups and the adult males are assessed for serum levels for thyroid hormones (T4). Further assessment of T4 in blood samples from the dams and day 4 pups is done if relevant. As an option, other hormones may be measured if relevant. Pup blood can be pooled by litter for thyroid hormone analyses. Thyroid hormones (T4 and TSH) should preferably be measured as 'total'.

[1] Hematology: hematocrit, hemoglobin concentrations, erythrocyte count, reticulocytes, total and differential leucocyte count, platelet count and a measure of blood clotting time/potential. Other determinations that should be carried out, if the test substance or its putative metabolites have or are suspected to have oxidizing properties include methemoglobin concentration and Heinz bodies.

[2] Clinical biochemistry: Overnight fasting of the animals prior to blood sampling is recommended. Investigations of plasma or serum should include sodium, potassium, glucose, total cholesterol, urea, creatinine, total protein and albumin, at least two enzymes indicative of hepatocellular effects (such as alanine aminotransferase, aspartate aminotransferase and sorbitol dehydrogenase) and bile acids. Measurements of additional enzymes (of hepatic or other origin) and bilirubin may provide useful information under certain circumstances

Reference

1. OECD. *Test No. 422: Combined Repeated Dose Toxicity Study with the Reproduction/Developmental Toxicity Screening Test*; 2025.
